# Structure of the Small-pox Pustule

**Published:** 1842-09

**Authors:** 


*Structure of the Small-pox Pustule.*—Mr. W. H. Judd has communicated, in the *Lancet* (June 12, 1841), some interesting observations on the structure of the small-pox pustule. Mr. J. has dissected the skin whilst it was under the influence of variola, in its various stages, and has discovered, he states, a very extraordinary septum and band in the small-pox pustule. The eruption, he remarks, commences by little red points on the skin resembling flea-bites. This appearance Mr. J. found "to arise from increased vascularity, caused by zones of minute vessels enlarging and projecting from the surface of the cutis; they secrete a thin serum, which gradually raises a ring of the cuticula externa from the rete mucosum, and so forms a vesicle without breaking up some of the thread-like attachments and ducts in the centre, between the cutis, rete, and cuticle. Hence the cuticular covering of the vesicle is bound down at this spot by that thread-like band, which causes it to have a peculiar depressed summit. As the disease advances, the efflorescence and inflammation increasing, the serum becomes gradually more opaque, and coagulable lymph is next thrown out, which at once consolidates and forms a thin flat layer, or plate, shaped like a cymbal, but with a small hole left through its centre, from the circumstance of the coagulation taking place around the before mentioned thread-like attachment of the cuticle. About this period the fever and inflammation are increased (called the secondary or suppurative fever); and in this stage pus being secreted instead of lymph, it elevates the lately described cymbal-like plate, and causes it to divide the cavity horizontally into an upper and lower cell. As the disease advances, the progressive distension breaks up the remaining attachment between the cuticle and cutis: the pus in the cell below passes through the hole in the cymbal-like plate, or septum, above, and blends with the serum in the vesicle, changing it into a pustule (called the maturing stage). By this time the lower part of the pustule is completed by an extremely thickened state of the rete mucosum, which forms a raised lip, or cup, constituting its base; so as, in ordinary cases, to shut off and protect the cutis from the contact of pus and ulceration. Hence, in most instances, the pustule may be stripped off with the cuticle and thickened rete, leaving the cutis entire; but the cutis vera has frequently a slight depression, or pit, left from the effect of the ulceration which has penetrated the base of the cup; and occasionally a small papula or two of the cutis is found projecting into its centre, to which the thread-like band of attachment from the cuticle still adheres."

Mr. Judd next describes the decline of the pustule and its consequences. "About the eleventh day (varying a day more or less in different epidemics) the cuticular covering bursts a little to one side of the apex of each pustule, and its contents gradually ooze out. If the affection has run through its stages mildly, the chief part of the fluid evacuated is but opaque lymph, which drying, forms a dark crust; if, on the contrary, the disease has been severe or confluent, an offensive pus is let out, which leads to scabs, sores, and ulcers in various parts. In bad and confluent cases, where inflammation has run high, not only is pus formed in contact with the cutis at the base of each pustule, but there are also thrown off shreds or sloughs from the true skin, causing permanent depressions, or white pits, for life. Temporary red stains are always left upon the skin, for a time after this eruption, and are caused by increased vascularity, thickening and rising of the rete; but these disappear if no ulceration takes place.

"Having anatomically traced the cause, we are now fully prepared to enter upon the second stage of our inquiry; viz. the prevention of the effect, or pits, and consequent deformity. In the first place, I shall premise the propriety of lessening, on the attack, the attendant fever and heat by all prudent means; as the height to which the edges of the cup, or scar, will rise, and the depth to which ulceration, or slough, will proceed, depend in a great measure upon the degree of inflammation and fever (though some families are certainly also influenced by constitutional peculiarity;*) but, commonly, I cannot advise blood-letting, especially to any extent, on account of the debility that invariably follows this disease; nor can I praise the use of antimonials and medicines that determine to the skin, as by increasing the cutaneous circulation they give rise to a fuller development of the pustules. I believe the safest and best mode of treatment is to give a brisk cathartic of calomel and jalap; to keep the patient on a strictly antiphlogistic diet; to put out the fire and lighten the clothing, so as to keep the sufferer's temperature as near the standard of health by the thermometer as possible, yet not so low as to impede a fair development of the eruption on most parts of the body. Nor must we abstract food, for so many days in succession, as to let the system flag from that cause; for a certain degree of vigor is needful: but all beyond this does harm. Just after maturation the patient will require support; and in some cases, meat, wine, or even bark. I should not have touched upon treatment, had not the size of the pustule, and consequent extent of the pit and deformity that will remain after the eruption, so much depended on it: and also the prevention of cerebral symptoms, sometimes said to occur on interfering, or suppressing, much of the general eruption.

"I shall next remind the reader, that the face, arms, and parts commonly exposed to light and air, always, as we might anticipate, suffer most deformity by small-pox pits; for air and light give vigor to arterial action, darkly tint the leaf, and heightens color in the cheek and pits. Hence small-pox pustules on the tongue, throat, and parts excluded from their agency, are nearly white, and seldom fully developed; and hence the success of Dr. Picton's method of preventing the ulcerative process, pits and stains, by the exclusion of light

---

* As is more fully described in my treatise on Syphilis, p. 122.

and air from the chamber of the sick. But in doing this the opposite extreme must be avoided; for it is well known that warmth and moisture (for you can scarcely exclude the air without increasing warmth and moisture) increase suppuration, and materially influence the development of all eruptions: even a poultice applied to a vaccine *vesicle,* from its warmth and moisture, will so alter the action of its secretory vessels, as in twenty-four hours to convert it into a *pustule.* Destroying the pores and the transparency of the skin by the application of nitrate of silver, successfully practised by Dr. Serres in this disease, amounts, in my opinion, only to the exclusion of light and gases.

"Mr. Le Grand's plan of smearing the surface with gum-mucilage, and covering with gold beater's skin, also lessens the action of light and excludes air; but it advances a step farther, by exerting another powerful curative agency, viz. an uniform pressure. Which reminds me of a method I have pursued for years, that of curing pustules, vesicles and sloughing ulcers on the face, &c., by a stiff coating of hot yellow wax and oil, after they had resisted all common treatment.

"Mr. George's plan of caking over the surface with calamine, acts not only by excluding light, but by absorbing moisture, and through its astringent qualities causing contraction of the living pustule. I have long since so applied the white oxide of zinc, and found it to act in a similar way upon vesicles, causing them to break, part with their contents, and heal. A coating of pure liquor plumbi acts in almost the same manner; and many eruptions about the face, hands, and neck, that have been obstinate, cease to come out, and speedily heal, if light and air be excluded, and motion restrained by uniform pressure; though effected if only by adhesive plaster and a roller.* Thus we approach by degrees, and in effect, almost to the method recommended by Dr. Olliffe; viz. the application of emplastrum ammoniaci cum hydrargyro, which in itself seems to possess the curative qualities of most of the other methods, with the valuable addition of power to promote absorption of the serum and lymph, when applied early in the disease, or even of the raised edges of the cup-like bases, which give rise to pits, scars and seams in a later stage. If the serum and lymph be thus caused to be absorbed, the vesicles are not completed, and therefore the pustules do not

---

* I have not noticed the early puncturing of each pustule, which is useful to prevent pits, and but anticipate Nature's plan.

occur. And this power over the disease we may fairly consider the remedy to possess, knowing, as we do, that the absorption of new-formed parts can be so much more easily promoted than that of originally formed structure. Any other specific action from the mercury and ammoniacum appears very unlikely; and as to animalcula causing the pits, Dr. Olliffe may certainly dismiss that idea from his consideration, notwithstanding that animalcules exist in all long-retained secretions in the human body.

After numerous dissections and microscopic examination, I may affirm that this eruption does not commence by enlarged papillæ, but by simple zones of minute vessels projecting from the cutis, throwing out serum, and raising the vesicle: and that although the red depositions protrude beneath the cuticle, so as to be felt on the surface, it is merely from distension; and enlarged papillæ are not to be found until after the maturating stage, and not then, except in the base of the pustule, and only when ulceration has penetrated the surface of the cutis. In many cases, even where lasting pits and deformity ensue, sloughing and ulceration will not generally be found to have penetrated through the corium to the cellular membrane beneath. I explain these points merely to set right some mistaken theory in the lately published paper.

"The application of the mask, or plaster, in the very early part of the eruption, is well insisted on. It should be applied as soon as the nature of the eruption is ascertained, and the red projections can be distinctly felt as early as the third day, and be continued *without intermission or removal* until the maturating stage has been completed in other parts of the body, a period of about five days. That this mode of treatment possesses vast power to arrest the full development of the eruption is undoubted; and I conceive so innocent a preventive merits trial by all medical practitioners desirous of preserving the appearance of their patients, and of advancing science."—*Am. Jour. Med. Sci.*

